# Adaptive immunity in different CT patterns of active tuberculosis and possible variability according to patients' geographic provenience

**DOI:** 10.3389/fmed.2022.890609

**Published:** 2022-09-07

**Authors:** Giulia Scioscia, Donato Lacedonia, Ernesto Giuffreda, Incoronata Caccavo, Carla Maria Irene Quarato, Piera Soccio, Pasquale Tondo, Ennio Vincenzo Sassani, Dalila Pescatore, Maria Pia Foschino Barbaro

**Affiliations:** ^1^Department of Medical and Surgical Sciences, University of Foggia, Foggia, Italy; ^2^Institute of Respiratory Diseases, Policlinico Riuniti of Foggia, Foggia, Italy; ^3^Internistic Department, Institute of Respiratory Disease, Azienda Ospedaliera Regionale San Carlo, Potenza, Italy; ^4^Institute of Radiology, Policlinico Riuniti of Foggia, Foggia, Italy

**Keywords:** tuberculosis, adaptive immunity, flow cytometry, CT pattern, geographic provenience

## Abstract

**Background:**

It is still unclear if low lymphocyte levels are directly related to immunological modifications induced by the TB infection or if they depend on the general pre-existing health impairment of affected patients. Our aim was to detect eventual differences in the immunological status of patients with pulmonary TB compared to an age and sex-matched group of hospitalized patients with other bacterial community-acquired pneumonia (CAP). In addition, we tried to assess an association between alterations in the peripheral lymphocyte subsets and the development of different CT patterns of active TB and to discover differences in the immunological status and in the radiological patterns of TB presentation between patients of different geographic proveniences.

**Methods:**

This observational study included 48 patients with TB and 48 sex- and age-matched patients affected by other bacterial CAP. The presence of HIV/AIDS, other immunocompromising conditions, and confounding chronic pulmonary comorbidities was excluded. Flow cytometry was performed on all the enrolled subjects at admission, before starting the appropriate antibiotic therapy. Patients with TB also underwent a computed tomography (CT) scan.

**Results:**

Patients with TB showed a decrease in the absolute count of all the lymphocyte subsets compared to the CAP group. Only the reduction in the percentage of CD4+ T-lymphocytes was significant, while the percentage of CD8+ T-lymphocytes was significantly increased. Patients presenting exudative forms with atypical locations of TB showed a significant reduction in the absolute count and percentage of CD19+ B-lymphocytes compared to those affected by productive TB forms with the typical location. Despite being younger, our black Sub-Saharan Africans showed a significant reduction in the CD4+ T-lymphocytes compartment and a higher prevalence of atypical and exudative forms of TB compared with white Europeans.

**Conclusion:**

Tuberculosis itself may alter peripheral blood lymphocyte subsets compared to other CAP. An impaired CD19+ B-lymphocyte compartment may result in an abnormal exudative response in atypical locations and a suboptimal bacterial control. Other constitutive or environmental causes may influence immunological differences found in patients with TB, particularly in case of different geographic origins. Anyhow, flow cytometry may be of great value in evaluating the immune function of these patients.

## Introduction

Despite the availability of specific chemotherapy since the 1950s has contributed to drastically reducing the number of cases (1), the WHO Global Tuberculosis Report of 2021 still defines tuberculosis (TB) as one of the top causes of death from a single infectious agent worldwide (ranking above HIV/AIDS) (2). The main reasons for the “re-emergence” of TB are the increased onset of socio-economic disparities related to migratory flow from developing countries and the increase in immunosuppressive diseases or, anyhow, diseases requiring immunosuppressive treatments (3).

Host defense mechanisms play a key role in determining the clinical course and the eventual different manifestations of such infection (4). After the inhalation of tubercle bacilli, several scenarios may follow. Inhaled bacilli may be trapped in the upper respiratory airways and expelled by the mucociliary clearance or may reach the alveoli being engulfed by alveolar macrophages in phagosomes and destroyed. Sometimes, a part of bacilli manages to escape from this killing mechanism, replicates within the phagocytic cells, and leads to their destruction. The consequent production of inflammatory cytokines and chemokines results in the in-site recruitment of additional macrophages and neutrophils, which, once again, try to engulf mycobacteria without destroying them. About 2–3 weeks after infection, a T-cell response (both CD4+ and CD8+) leads to the granuloma formation (5). The tuberculous granuloma is an organized structure, formed by specialized macrophages harboring and surrounding the mycobacteria at the center and a rim of lymphocytes, that acts as a barrier to preventing the spread of infection. Over time, the granuloma usually undergoes a productive reaction, in which prevails the formation of a special granulation tissue tending to transform into fibrotic tissue and to heal by scarring (6, 7).

Immunosuppressive conditions, involving particular cell-mediated immunity or a tumor necrosis factor alfa (TNF-α) deficiency, can limit the formation of a well-organized granuloma. This results in the persistence of an exudative reaction (i.e., liquid material in the interstitial space with a tendency to develop caseous necrosis), favoring, therefore, the spread of infection and the development of active disease (7, 8). A subsequent state of immune suppression can also provoke the re-activation of the disease starting from bacillary elements that eventually remained “walled-up alive” inside a granuloma, being, therefore, capable of resuming their virulence (6). Malnutrition, with the consequent immune deficits that derive from it, as well as poor hygienic-sanitary and socio-economic conditions, represent particularly relevant risk factors for re-infection, especially in the population of immigrants (8).

Based on the evolution of the pulmonary lesions, it is radiographically possible to distinguish between mainly “productive” and mainly “exudative” TB forms (9, 10). Productive TB is characterized by more or less extensive fibrous and retracted areas with circumscribed and calcified caseous centers (nodules) or frank cavity lesions with strongly thickened walls (caves). These lesions typically affect the apical and posterior segments of the upper lobes or the upper segments of the lower lobes (10). Exudative radiological manifestations are lobar or segmental consolidations (tuberculous pneumonia) and pleural effusions (9) frequently involving atypical locations, such as the lower segments of the lower lobes, the middle lobe, the lingula, and the anterior segment of the upper lobes. These forms may also be multifocal (11).

The aim of this study was to evaluate the immunological status of patients with active pulmonary TB compared to an age and sex-matched group of hospitalized patients with other bacterial community-acquired pneumonia (CAP). Additionally, we tried to assess the possible association between an eventual alteration in the circulating lymphocyte subsets and the development of different radiological patterns of active TB (i.e., productive forms with typical location or exudative forms with atypical locations) and whether a different geographic provenience may influence the variability in the immunological status and in the radiological patterns of presentation of TB.

## Materials and methods

### Participants

This observational case-control study was carried out in the Institute of Respiratory Diseases of the University Polyclinic “Riuniti” of Foggia from January 2016 to January 2020. Our population consisted of 48 patients with TB (35 men and 13 women; mean age ± SD 42.06 ± 19.11 years) and of 48 sex and age-matched patients affected by other bacterial community-acquired pneumonia (CAP) and admitted to our hospital in the same period of time. The diagnosis of CAP was based on clinical signs and symptoms and the presence of pulmonary infiltrates on the chest radiograph at admission. The diagnosis of TB was confirmed by positive cultures for *Mycobacterium tuberculosis* from sputum or bronchoalveolar lavage specimens. Patients affected by HIV/AIDS and other immunocompromising conditions, namely, diabetes mellitus, chronic kidney disease, a history of hematologic or solid-organ neoplasm, and rheumatologic diseases on immune-modulatory therapy, were excluded from the study. Similarly, we excluded patients affected by other chronic pulmonary conditions (i.e., chronic obstructive pulmonary disease, pulmonary fibrosis, bronchial asthma) that could produce alteration in the various circulating lymphocyte subsets. A flow cytometry study was performed on all the patients before receiving the appropriate treatment. Patients with TB also underwent a computed tomography (CT) scan.

This study was carried out according to the principles of the Declaration of Helsinki, and was approved by the local ethics committee of the University Polyclinic “Riuniti” of Foggia (institutional review board approval N° 17/CE/2014), and all recruited patients gave their written informed consent.

### Flow cytometry

Peripheral venous blood samples (5 mL) were collected into tubes with EDTA from all the subjects in the study (patients with TB and CAP) before receiving the appropriate antibiotic therapy. Absolute counts and percentages of lymphocyte subsets were determined using the Becton, Dickinson, and Company (BD) Multi-test 6-color direct TBNK immunofluorescence reagent with BD Trucount tubes (BD Biosciences, USA). Briefly, 50 μl of whole blood was dispensed into an ID-labeled Trucount tube and incubated for 15 min in the dark at room temperature with 20 μl of TBNK reagent, containing the following mixture of fluorochrome-conjugated mAbs: anti-CD45-peridinin chlorophyll protein-Cyanine5.5 (PerCP-Cy5.5), anti-CD3-fluorescein isothiocyanate (FITC), anti-CD4-phycoerythrin-cyanine7 (PE-Cy7), anti-CD8-allophycocyanin-cyanine7 (APC-Cy7), anti-CD19-allophycocyanin (APC), and anti-CD16+CD56-phycoerythrin (PE). At the end of the incubation time, 450 μl of BD FACS Lysing Solution (BD, Biosciences) was added to each tube, and tubes were incubated again for 10 min at room temperature in darkness. Stained samples were finally processed in a BD FACSCanto flow cytometer using BD FACSCanto clinical software version 2.0 (BD Biosciences, USA). According to the Producer Company of the mixture of fluorochrome-conjugated mAbs used, normal reference ranges in terms of absolute count and percentage for total lymphocytes and their differential subsets were the followings: CD45+ total lymphocyte−1000.0–4500.0 cells/μL; CD4+CD3+ helper T-lymphocytes−491–1734 cells/μL (31.45–62.38%); CD8+CD3+ suppressor T-lymphocytes−162–1074 cells/μL (9.55–38.32%); CD19+ B-lymphocytes−73–562 cells/μL (5.89–24.21%); CD16+56+ NK cells−108–680 cells/μL (5.17–30.36%); CD4+/CD8+ ratio (0.50–2.74).

### Chest CT patterns

A chest CT scan was obtained in patients affected by TB. We divided chest CT findings into exudative forms (pneumonia and pleural effusion) and productive forms (cavitary or nodular lesions) (9, 11). According to the topography of lesions, we classified chest CT findings in typical localization [apical and posterior segments of the upper lobes and superior segment of the lower lobes (9, 10)] and atypical localization [anterior segment of the upper lobes, middle lobe/lingular involvement, inferior segments of the lower lobes, or multifocal localization (9, 11)].

### Statistical analysis

Results are expressed as mean ± standard deviation (SD) or median with first and third quartile (Q1;Q3) for continuous variables and as count (c) and percentage (%) for descriptive variables. Differences in terms of lymphocyte subsets between the CAP and the TB group were tested for significance using the Mann–Whitney U test. The chi-square test (χ2) was used to assess significant differences in terms of descriptive variables. A *p*-value of < 0.05 was considered to be significant. The strength of the association between TB or CAP and the reduction in the absolute count of the different lymphocyte subsets below normal ranges were estimated in terms of odds ratios (OR) with a 95% confidence interval (CI). A subgroup analysis was performed among patients with TB by diving the enrolled subjects in two groups according to their radiological pattern (typical/productive or atypical/exudative forms) and ethnicity (black foreign-born people coming from Sub-Saharan Africa or white foreign-born people coming from East Europe and native Italian). The relationship between the different radiological patterns of TB and ethnicity was investigated by calculating the odds ratios with 95% confidence intervals.

### Comparison between patients with TB and CAP

Demographic characteristics and flow cytometry results of patients enrolled in the TB group and in the control group are shown in Table 1.

**Table 1:** Demographic characteristics and flow cytometry results in the control group and in the TB group.

	**CAP group (*n* = 48)**	**TB group (*n* = 48)**	***p*-value**
Age, mean ± SD	42.06 ± 19.11	42.06 ± 19.11	1.0
**Sex**
Male, c (%)	35 (73%)	35 (73%)	*^χ^* 1.0
Female, c (%)	13 (27%)	13 (27%)	
**Geographical origin**
White–Italia	41 (85%)	21 (44%)	*^χ^* <0.0001*
White-Southeast Europe^a^	0 (0%)	8 (17%)	*^χ^* 0.006*
Black–West Africa^b^	6 (13%)	15 (31%)	*^χ^* 0.046*
Black – Central Africa^c^	1 (2%)	4 (8%)	*^χ^* 0.4
**Flow cytometry, median (Q1; Q3)**
TL, c (cells/μL)	1413.1 (1262.0; 1881.8)	961.7 (676.8; 1371.3)	<0.0001*
CD4+, c (cells/μL)	913.3 (752.2, 1162.4)	490.0 (417.3, 795.1)	<0.0001*
CD4+, %	46.26 (39.35; 52.98)	42.28 (37.52; 46.98)	0.03*
CD8+, c (cells/μL)	463.4 (349.9; 645.9)	354.9 (219.3; 533.4)	0.02*
CD8+, %	21.80 (16.83; 28.78)	27.30 (20.88; 32.25)	0.02*
BL, c (cells/μL)	165.2 (108.5; 257.6)	113.7 (59.4; 206.1)	0.001*
BL, %	7.9 (5.6; 11.7)	8.6 (6.0; 12.2)	0.62
NKL, c (cells/μL)	303.1 (193.1; 523.0)	187.4 (92.66; 300.9)	0.0009*
NKL, %	15.1 (10.4; 23.6)	13.7 (8.1; 19.7)	0.38
CD4+/CD8+	1.98 (1.59; 3.05)	1.56 (1.23; 2.05)	0.0096*

CAP, community-acquired pneumonia; TB, tuberculosis; TL, total lymphocytes; BL, B-lymphocytes, NKL, natural killer lymphocytes; SD, standard deviation; Q1, first quartile; Q3 third quartile. ^*a*^Southeast Europe: patients coming from Bulgaria, Romania, and Serbia; ^*b*^West Africa: patients coming from The Gambia, Ghana, Nigeria, Senegal, Sierra Leone, and Togo; ^*c*^Central Africa: patients coming from Cameroon, the Democratic Republic of the Congo, Gabon. (^χ^) Assessed with chi-square test. p < 0.05 is denoted by ^*^.

There was no difference in age and gender among the subject enrolled in the two groups. TB cases were detected in 27 foreign-born people and 21 native Italian. Among the foreign-born people with TB included in the study, eight were white people coming from East Europe and 19 were black people coming from Sub-Saharan Africa.

Patients with TB showed a lower number of total lymphocytes and a decrease in the absolute number of CD4+ T-lymphocytes, CD8+ T-lymphocytes, B-lymphocytes, and NK cells compared to patients affected by other bacterial CAP. However, only the reduction in the percentage of CD4+ T-lymphocytes was significant, while the percentage of CD8+ T-lymphocyte was significantly increased. The CD4+/CD8+ ratio was significantly reduced in TB cases compared to CAP.

Table 2 shows the odds ratios of finding an absolute number of the different lymphocyte subsets below normal ranges in the two groups.

**Table 2:** “Odds ratios of finding an absolute number of total lymphocytes and their subpopulations below normal ranges in the TB group compared to the CAP group.”

	**CAP group (*n* = 48)**	**TB group (*n* = 48)**	**OR (95% CI)**	***p*-value**
TL <1,000 cells/μL	5 (10%)	24 (50%)	8.6 (2.9–25.5)	<0.0001*
CD4+ <491 cells/μL	1 (2%)	27 (56%)	60.4 (7.7-474.7)	<0.0001*
CD8+ <162 cells/μL	0 (0%)	6 (13%)	14.8 (0.8-271.2)	0.07
BL <73 cells/μL	6 (13%)	16 (33%)	3.5 (1.2-10.0)	0.02*
NK <108 cells/μL	3 (6%)	16 (33%)	7.5 (2.0-27.9)	0.003*

TB, tuberculosis; TL, total lymphocytes; BL, B-lymphocytes, NKL, natural killer lymphocytes; CI, confidence interval. p < 0.05 is denoted by *.

### Comparison between patients with TB with different CT patterns (exudative forms with atypical locations; productive forms with typical location)

Demographic characteristics and flow cytometry results of patients with TB with atypical/exudative and typical/productive forms are shown in Table 3.

**Table 3:** Demographic characteristics and flow cytometry results in patients with TB according to their chest CT pattern (productive forms with typical location or exudative forms with atypical locations).

	**Productive forms with typical location (*n* = 24)**	**Exudative forms with atypical location** **(*n* = 13)**	***p*-value**
Age, mean ± SD	41.23 ± 10.51	40.69 ± 13.27	0.77
**Flow cytometry, median (Q1; Q3)**
TL, c (cells/μL)	1368.0 (1113.0; 1510.6)	588.9 (438.7; 620.1)	<0.0001*
CD4+, *n* (cells/μL)	803.1 (591.9; 925.9)	382.9 (297.9; 440.8)	<0.0001*
CD4+, %	46.5 (37.06; 51.93)	42.74 (40.22; 46.33)	0.52
CD8+, c (cells/μL)	512.2 (329.9; 619.1)	171.1 (136.1; 237.4)	<0.0001*
CD8+, %	27.1 (19.54; 31.66)	26.22 (21.51; 32.00)	0.87
BL, c (cells/μL)	198.7 (115.9; 276.5)	56.2 (28.1; 78.8)	<0.0001*
BL, %	10.20 (6.65; 14.45)	7.25 (4.68; 9.63)	0.03*
NKL, c (cells/μL)	203.6 (151.0; 336.7)	83.4 (65.2; 130.7)	0.002*
NKL, %	13.54 (7.93; 19.13)	13.73 (7.91; 16.31)	0.83
CD4+/CD8+	1.47 (1.20; 2.87)	1.77 (1.48; 2.03)	0.37

TL, total lymphocytes; BL, B-lymphocytes, NKL, natural killer lymphocytes; SD, standard deviation; Q1, first quartile; Q3 third quartile. p < 0.05 is denoted by ^*^.

No difference was assessed in the mean age of patients belonging to the two groups. Patients with TB with exudative forms and atypical locations showed a lower count of CD4 + T-lymphocytes, CD8 + T-lymphocytes, B-lymphocytes, and NK cells than patients with TB with productive forms and typical location. However, the CD4+, CD8+, and NK cells absolute count significant difference was only linked to the total lymphocyte absolute count significant difference, as the percentages of these lymphocyte subsets were not significantly different. Only the reduction in the percentage of CD19 + B-lymphocytes assessed in exudative forms with atypical locations reached a statistical significance.

### Comparison between patients with TB with different geographical proveniences (Africans/Europeans)

Demographic characteristics and flow cytometry results in black TB patients coming from Sub-Saharan Africa and white TB patients coming from East Europe or native Italian are shown in Table 4. Black Sub-Saharan Africans were younger than white Europeans and showed a lower absolute count and percentage of CD4+ T-lymphocytes. In addition, black Sub-Saharan Africans showed a higher prevalence of exudative forms and atypical lesions' location than white Europeans.

**Table 4:** Demographic characteristics, flow cytometry, and chest CT results in patients with TB according to their nationality (Europeans or Africans).

	**White Europeans (*n* = 29)**	**Black Africans (*n* = 19)**	***p*-value**
Age, mean ± SD	48.83 ± 20.09	31.74 ± 11.82	0.0017*
**Flow Citometry median (Q1; Q3)**
TL, c (cells/μL)	1019 (830; 1433)	803 (602; 1161)	0.05
CD4+, c (cells/μL)	726 (444; 901)	472 (369; 536)	0.009*
CD4+, %	45.01 (38.86; 48.14)	38.71 (33.06; 45.77)	0.03*
CD8+, c (cells/μL)	356 (231; 556)	337 (182; 522)	0.45
CD8+, %	26.07 (19.59; 30.18)	31.63 (24.42; 33.49)	0.10
BL, c (cells/μL)	145 (68; 209)	94 (43; 123)	0.08
BL, %	8.90 (6.40; 12.00)	7.30 (5.05; 11.85)	0.41
NKL, c (cells/μL)	199 (131; 298)	140 (84; 284)	0.24
NKL, %	13.65 (8.14; 19.05)	13.73 (7.05; 21.48)	0.93
CD4+/CD8+	1.59 (1.31; 2.85)	1.48 (1.11; 1.85)	0.12
**CT pattern**
Productive forms	22 (76%)	8 (42%)	*^χ^* 0.03*
Exudative forms	7 (24%)	11 (57%)	
Typical	21 (72%)	7 (37%)	*^χ^* 0.02*
Atypical	8 (28%)	12 (63%)	

TL, total lymphocytes; BL, B-lymphocytes, NKL, natural killer lymphocytes; SD, standard deviation; Q1, first quartile; Q3 third quartile. (^χ^) Assessed with chi-square test. p < 0.05 is denoted by ^*^.

## Discussion

In our study, patients with TB showed global lymphocytopenia with a significantly lower count and percentage of CD4+T-cells in the peripheral blood than patients affected by other bacterial CAP. This finding accords with previous studies that suggested a decreased CD4+ T-lymphocyte count in the peripheral blood of patients with TB (12–20). Moreover, no significant differences were observed in the percentages of CD19+ B-lymphocytes and CD16+56+ NK cells between the TB and CAP groups, while the percentage of CD8+ T-lymphocytes was even increased. This data could be interpreted as indicating that the global reduction in the absolute count of total lymphocytes observed in our patients with TB is more pronounced for CD4+ T-lymphocytes.

It is generally accepted that CD4+ T-lymphocytes are an essential component of protective immunity against tuberculosis (21, 22). As a confirmation, patients with AIDS, which is characterized by a specific blood reduction of these cells, have a higher incidence and severity of tuberculosis (17, 23). Following infection, *M. tuberculosis* is internalized by macrophages and dendritic cells and trapped in a vacuole called a phagosome. Here, mycobacterial polypeptides are digested and the resulting antigenic peptide fragments are loaded onto major histocompatibility complex (MHC) Class II molecules through which they are presented to CD4+ T-cells. The main function of CD4+ T-cells in immunity against TB is to differentiate into T-helper type 1 (Th-1) effector cells and produce interferon-gamma (IFN-γ), which directly activates macrophages for controlling infection, and TNF-α, which induces apoptosis of macrophages infected with mycobacterium and guides the granuloma formation (4, 24). Peripheral CD4+ T-cell reduction in patients with TB has already been interpreted as a consequence of an augmented pooling of these cells at the site of infection. This hypothesis has been sustained by molecular and clinical studies (19, 25–27). Other authors have suggested an important shift toward the pulmonary local compartment to explain the transient lymphocytopenia observed during the acute phase of other bacterial CAP (28). Anyhow, we must not forget that TB itself may determine a further depletion in the CD4+ T-cells compartment. *M. tuberculosis* and its constituents are known to stimulate monocytes in producing an array of cytokines among which the transforming growth factor beta (TGF-β) is characterized by suppressive action on CD4+ T-lymphocytes (29, 30). Further evidence come from the finding of lower levels of CD4+ T-lymphocytes in HIV/TB subjects compared to subjects with only HIV (31). In addition, the decreased CD4^+^ T-cell counts in patients with TB have been shown to return to normal levels after the correct anti-TB chemiotherapy (12, 32, 33).

The role of CD8^+^ T-lymphocytes in the control of *M*. *tuberculosis* infection is still controversial. CD8+ T-lymphocytes are MHC Class I-restricted cells typically involved in cytotoxicity during adaptive immune responses. Like CD4+ T-lymphocytes, CD8+ cells are also capable of producing interleukin 2 (IL-2), IFN-γ, and TNF-α (34). MHC Class I molecules gain their peptide ligands primarily from the cytoplasm. Despite *M. tuberculosis* being usually found within the phagosomes of infected macrophages, either the bacteria, and the bacterial products can gain access to the cytosol (35). As a result, CD8+ T-cells are able to recognize infected cells and eliminate them through different mechanisms, such as the Fas–Fas ligand interaction, the TNF-mediated killing, and the release of perforin, granzyme, and granulysin by granule exocytosis. As a confirmation, CD8+ T-cells from patients with TB have been shown to exhibit greater cytotoxic activity than healthy donors (36). In addition, a subset of unconventional CD8+ T-cells has been identified that can recognize *M. tuberculosis*-specific antigens presented by a variety of other non-classical MHC I-like molecules, namely, MHC Ib and CD1a-d (37). Lipoarabinomannan (LAM) seems to be the most potent mycobacterial lipid antigen in activating a specific type of polycytotoxic CD1b-restricted CD8+ T-cells (38, 39). These LAM-responsive unconventional CD8+ T-cells co-express perforin, granzyme B, and granulysin and may have a key role in infection control (38). Available data on CD8+ T-lymphocytes count in patients with TB are contrasting. In some studies, the CD8^+^ T-cells count was significantly decreased (15, 16, 40, 41), while in other studies it was unmodified (13, 15) or increased (14, 32) compared to healthy controls. In our study, the finding of a significantly decreased CD4+/CD8+ ratio in patients with TB compared to patients with CAP might be explained by assuming both a peripheral depletion of the CD4+ T-cells compartment and an increase in the peripheral CD8+ T-cells population. This is evident from the CD4+ and CD8+ percentages in patients with TB compared to the CAP group.

Also, CD16+56+ NK cells and CD19+ B-lymphocytes may be found in the constitution of the TB granuloma (6). NK cells are capable of secreting IL-2, IFN-γ, and TNF-α without requiring any priming, thus representing bridge effectors between the innate and adaptive immune systems (42). During the early phase of *M. tuberculosis* infection, NK cells may provide an early source of IFN-γ that seems to be crucial for establishing the optimal proinflammatory cytokine milieu in shaping the T-cell response toward a Th-1-protective phenotype. NK cells can also exert cytotoxic activity against infected macrophages that are not well-equipped to control the intracellular growth of mycobacteria, with their consequent release into the extra-cellular space. This may allow NK cells to interact directly with whole bacteria or bacteria-derived products through the recognition of mycobacterial ligands by activating NK cell receptors (43, 44). B-lymphocytes can act as antigen-presenting cells that engulf antigens or whole mycobacterial bacilli and present them to T-cells in the granuloma. As a result, B-cells contribute to the induction of CD4+ T-cells responses to TB. In addition, B-cell antibodies may elicit effector functions on extra-cellular mycobacteria such as opsonization, which facilitates bacterial phagocytic uptake, antibody-dependant cellular cytotoxicity, and complement activation, which can further enhance opsonization and bacterial lysis, but also phagocytosis through complement receptors (45). Very few data are available in the literature about CD16+56+ NK cells and CD19+ B-lymphocytes count in patients with TB (46). In our study, active TB was associated with significant positive odds ratios in finding an absolute count of CD4+ T-lymphocytes, CD19+ B-cells, and CD16+56+ NK cells below normal ranges compared to other bacterial CAP. Only the odds assessing an actual circulating depletion for the CD8+ T-cells compartment did not reach a statistical significance. As no statistically significant difference was detected in the percentage distribution of CD19+ B-lymphocyte and CD16+56+ NK cells between the TB and the CAP group, we can speculate that the achievement of statistical significance only in the absolute count of these lymphocyte subsets is probably affected by a numeric reduction in peripheral blood subsequent to their pooling in the infected lung parenchyma.

**Figure 1:**
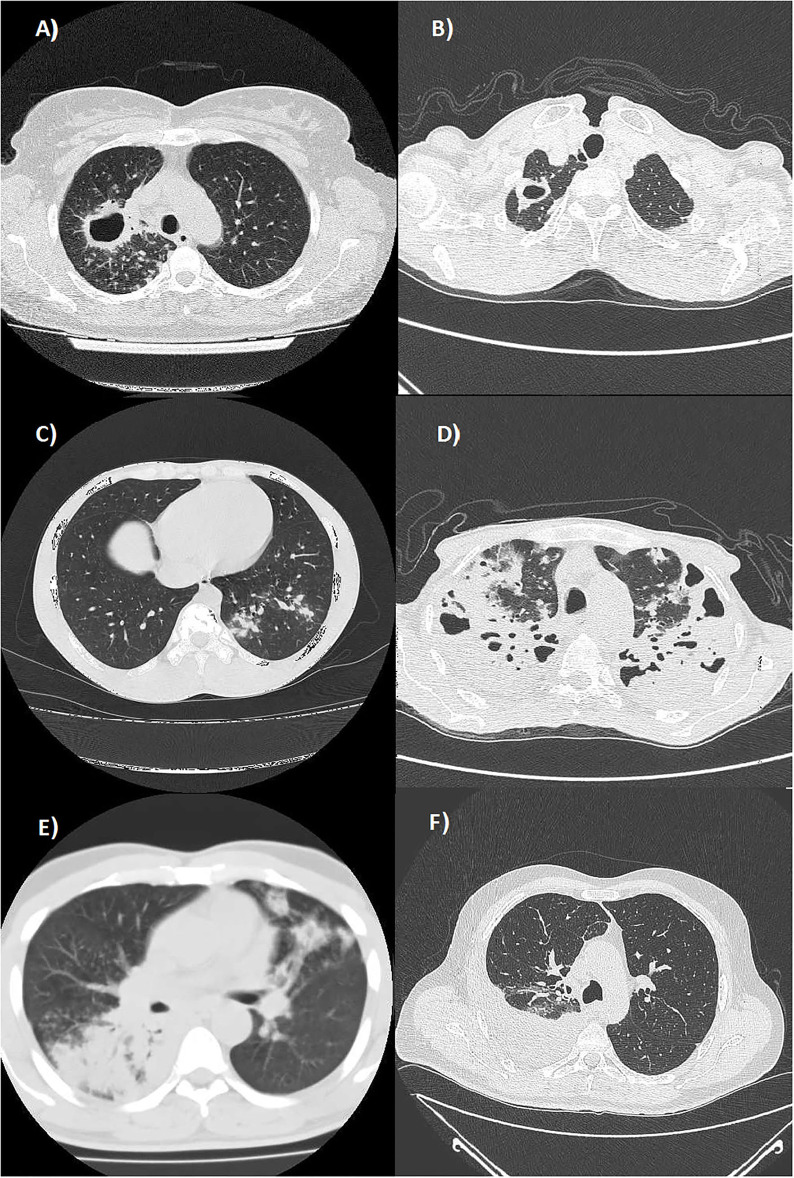
Axial Chest CT scans (lung window) showing: **(A)** multiple well-defined nodules diffusely distributed in the upper lobes and a cavitated lesion with thickened walls in the posterior segment of the right upper lobe (productive form with typical location); **(B)** a cavitated lesion with thickened walls in the apical segment of the right upper lobe (productive form with typical location); **(C)** a nodular pattern in the posterior segment of the left lower lobe (productive form with typical location); **(D)** a multifocal bilateral tubercular pneumonia in the upper lobes (exudative form with atypical locations); **(E)** multifocal tubercular pneumonia affecting the superior segment of the right lower lobe and the lingula (exudative form with atypical locations); and **(F)** a right tuberculous pleural effusion (exudative form with atypical locations).

Patients with TB affected by exudative forms with atypical locations showed a greater lymphocytopenia with a lower absolute count in all the lymphocyte subsets compared to patients with TB with productive forms and typical locations. In exudative TB forms with atypical locations, the reduction in either the absolute count or percentage reached a statistical significance only for the CD19+ B-lymphocytes. These data suggest that the coordinated response of the humoral and cellular adaptive immune system against *M. tuberculosis* may have a key role in the development of those encapsulating and fibrotic productive reactions giving rise to nodular or cavitary lesions that typically involve the apical and posterior segments of the upper lobes and in the upper segments of the lower lobes. Alternatively, an impaired humoral immunity may lead to a greater prevalence of exudative processes characterized by liquid and corpuscular exudation and modest connective reaction that frequently involve the lower segments of the lower lobes, the middle lobe, the lingula, and the anterior segment of the upper lobes or have a multifocal involvement (Figure 1). In this regard, studies on mice suggested that CD19+ B-lymphocytes may regulate the level of granulomatous reaction, cytokine production, and T-cell response (47). Other studies are needed to establish the role of reduced B-cell counts in patients with TB.

The current TB epidemiological situation in Italy is characterized by a low incidence in the native population, with a concentration of cases in risk groups, such as immigrants. In this study, the majority of TB cases affecting foreign-born people were detected in black Sub-Saharan Africans. These subjects showed a statistical reduction in the total count and percentage of CD4+ T-lymphocytes compared to white Italian and other European patients with TB, despite they were younger compared to the latter. In the absence of further predisposing factors for immunosuppression (i.e., enrolled subjects were HIV-negative and free from immunosuppressive diseases, as exclusion criteria), TB cases in black Sub-Saharan Africans might represent forms of newly acquired infection or reactivation, favored either by a state of malnutrition or by poor hygienic-sanitary and socio-economic conditions (i.e., urban overcrowding and poverty). Anyhow, this suggestion does not fully explain the higher prevalence of atypical/exudative forms in this TB population. This difference in immune responses might be mostly genetic/inherited from ancestors. According to the US Centers for Disease Control and Prevention (CDC), the TB case rate is eight times higher for Black or African American persons than for non-Hispanic White persons (48). A possible explanation is that white people of European descent are characterized by a more effective immune response to TB infection compared to black people of Sub-Saharan African descent. It is known that European emigrants brought Koch's bacillus to America. The same must be said of other countries which in the past, before the arrival of the Europeans, were totally free from the disease, while today are noted as areas of endemic TB, such as Sub-Saharan Africa. As a result, the less advantageous exudative response detected in Sub-Saharan Africans might be the result of a less effective natural selection in an environment in which there were fewer mycobacteria compared to Europe. Further genetic and molecular studies are required to confirm this suggestion.

Several limitations should be noticed in our study. First, the number of patients with TB enrolled was small. Indeed, our dedicated ward for respiratory diseases is located in a South Europe country with low TB incidence [in 2020, the incidence of tuberculosis in Italy was 6.6 cases per 100,000 people (49)], which makes it more difficult to collect a large number of patients. However, we were able to collect a number of patients that is comparable with other similar studies on the topic (13, 16, 17, 32, 41). Second, the analysis did not include a control group of healthy subjects for comparison. That is why this study was the result of observations made on a population of hospitalized patients requiring medical care. Anyhow, we tried to overcome this limitation by comparing the absolute count in the different lymphocyte subsets assessed in both the TB and CAP to the lower limits of the respective standard reference ranges in terms of the odds ratio. Third, the relatively small sample size in this study did not allow to pre-establish an ideal sample size for the subgroup analyses targeted for different radiological patterns (typical/productive or atypical/exudative forms) and ethnicities (black Sub-Saharan Africans or East European). Furthermore, “Africans” and “Europeans” subjects included in this study are not clearly entirely representative of the highly heterogeneous groups of people who originated in these two different continents. Despite these limitations, we believe that our suggestions could represent interesting starting points for further studies evaluating the potential interaction of active tuberculosis with the host's immune system.

## Conclusion

The reduction in the absolute count of lymphocyte subsets in our patients with TB may suggest increased compartmentalization at the site of infection compared to other bacterial CAP. Such depletion of circulating lymphocytes was more pronounced for the CD4+ T-cells subset, as it was evident from the CD4+ T-cells absolute count and percentage observed in patients with TB compared to the CAP group. On the contrary, the percentage of CD8+ T-lymphocytes was increased, influencing the statistically significant reduction of the CD4+/CD8+ ratio. Interestingly, exudative forms with atypical locations of pulmonary TB were associated with a significantly lower absolute count and percentage of CD19+ B-lymphocytes. This association may explain an abnormal granulomatous response resulting in suboptimal bacterial control. Finally, it should be noted that our black Sub-Saharan African patients, although younger, exhibited greater peripheral impairment in the CD4+ T-lymphocytes compartment and a greater prevalence of exudative and atypical forms of TB compared to white European patients with TB. The nutritional status of our patients has not been objectively evaluated in our study. Similarly, a molecular and genetic study assessing a different host's immune response has not been performed. In this regard, future studies are required to evaluate the potential interactions between *M. tuberculosis* and eventual underlying constitutive and environmental factors in depressing the immune system. Despite our suggestions needs confirms, examining lymphocyte subsets in patients with TB may be of great value in evaluating the immune function of these patients.

## Data availability statement

The raw data supporting the conclusions of this article will be made available by the authors, without undue reservation.

## Ethics statement

The studies involving human participants were reviewed and approved by Local Ethics Committee of the Policlinico Universitario Riuniti of Foggia (Institutional Review Board Approval N° 17/CE/2014). The patients/participants provided their written informed consent to participate in this study.

## Author contributions

MF was the guarantor of the content of the manuscript and including the data and analysis. GS, DL, EG, IC, and MF contributed to the conception and design of the study. DL, EG, IC, CQ, PS, PT, ES, and DP contributed to the acquisition, the analysis, and the interpretation of data. All the authors contributed to drafting the work, revising it critically, read, approved the final version of the paper, and agree to be accountable for all aspects of the work in ensuring that questions related to the accuracy or integrity of any part of the work are appropriately investigated and resolved.

## Conflict of interest

The authors declare that the research was conducted in the absence of any commercial or financial relationships that could be construed as a potential conflict of interest.

## Publisher's note

All claims expressed in this article are solely those of the authors and do not necessarily represent those of their affiliated organizations, or those of the publisher, the editors and the reviewers. Any product that may be evaluated in this article, or claim that may be made by its manufacturer, is not guaranteed or endorsed by the publisher.
